# Loss of BAP1 expression is associated with genetic mutation and can predict outcomes in gallbladder cancer

**DOI:** 10.1371/journal.pone.0206643

**Published:** 2018-11-05

**Authors:** Takashi Hirosawa, Masaharu Ishida, Kentaro Ishii, Keigo Kanehara, Katsuyoshi Kudo, Shinobu Ohnuma, Takashi Kamei, Fuyuhiko Motoi, Takeshi Naitoh, Florin M. Selaru, Michiaki Unno

**Affiliations:** 1 Department of Surgery, Tohoku University Graduate School of Medicine, Sendai, Miyagi, Japan; 2 Department of Medicine, Division of Gastroenterology and Hepatology, Johns Hopkins University, Baltimore, Maryland, United States of America; University of South Alabama Mitchell Cancer Institute, UNITED STATES

## Abstract

**Background:**

BRCA-1 associated protein (BAP1) is a de-ubiquitinating enzyme that regulates gene expression. Recently, the *BAP1* mutation and its involvement in cancer survival have been reported in a range of tumor types, including uveal melanoma, mesothelioma, renal cancers, and biliary tract cancers. However, the frequency of BAP1 mutation and down-regulation varies among tumor types, and little is known about the function of BAP1 silencing in cancer cells. Gallbladder carcinoma (GBC) is a type of biliary tract cancer with a poor prognosis. Few mutational studies have investigated the role of *BAP1* in GBC, and no functional study *in vitro*-, or clinical studies about cancer survival have been done.

**Methods:**

GBC cells were studied by following the small interfering RNA mediated silencing of BAP1 with regard to proliferation, migration, invasion, and drug sensitivity. We carried out genomic, epigenomic and immunohistochemical analyses to detect somatic *BAP1* alterations in 47 GBC patients undergoing surgical resection.

**Results:**

*BAP1* depletion resulted in increased migration and invasion, but not proliferation, and also resulted in decreased sensitivity to bortezomib, a proteasome inhibitor. Suppressed expression of *BAP1* occurred in 22 GBC cases (46.8%) and showed a strong trend toward a worse median survival time of 13.3 months (95% CI, 17.6–62.6) (p = 0.0034). Sanger sequencing revealed a loss-of-function mutation of *BAP1* in 11 out of these 22 GBC cases (50%) with low BAP1 expression, whereas 2 out of 25 GBC cases (8%) were detected in cases with high BAP1 expression. Partial changes in methylation were observed in 6 out of 47 cases, but methylation did not show a strong relationship to *BAP1* expression or to the prognosis.

**Conclusion:**

Our findings showed that genetic mutations are involved in *BAP1* down-regulation, leading to promotion of the invasive character of cancer cells and poor prognosis in GBC.

## Introduction

BRCA-1 -associated protein (BAP1) is a de-ubiquitinating enzyme (DUB), a member of the ubiquitin carboxyl-terminal hydrolase (UCH) subfamily, and is involved in cell cycle progression, gene transcription and DNA repair [1]. BAP1 was identified as a protein binding to the BRCA1 RING finger domain and is encoded by the *BAP1* gene at 3p21 [2]. No involvement of BAP1 in breast cancer has been found [3], however, and BAP1 has not been studied in the context of cancer for some time.

Recently, various mutations of *BAP1* have been found in several tumors, including uveal melanoma (UM) [4], mesothelioma [5], renal cell carcinoma (RCC) [6, 7], and intrahepatic cholangiocarcinoma (ICC) [8, 9], but the frequency of *BAP1* mutations varies widely among different tumor types. Van de Nes JA *et al*. [4] detected somatic BAP1 mutations in 50.7% of UM specimens (33 of 65), whereas Nasu M *et al* [5] detected mutations in 63.6% of mesotheliomas (14 of 22). Hakimi AA *et al*. [6] reported that *BAP1* showed mutations in 11 out of 185 clear cell RCC cases (5.9%). Simbolo M *et al*. [9] revealed *BAP1* mutations in 10 out of 70 ICC cases (14.3%) and 1 out of 26 cases (3.8%) of gallbladder carcinoma (GBC), whereas Jiao.Y *et al*. [8] revealed mutations in 8 out of 32 ICC cases (25%; discovery screen) and 1 out of 8 GBC cases (13%).

The germline *BAP1* mutation is associated with an increased risk of UM [10, 11, 12, 13], mesothelioma [11, 13, 14], cutaneous melanoma [11, 13], meningioma [12], RCC [13, 15] and MBAITs [11] (melanocytic *BAP1*-mutated atypical intradermal tumors) and is known as BAP1 hereditary cancer predisposition syndrome [13].

Gallbladder carcinoma is a biliary tract cancer derived from the gallbladder mucosa, and is a malignant disease with a poor prognosis, as is ICC [16]. The incidence of GBC is high in northern India, the Republic of Chile and Japan. The incidence in Japan is estimated at 7 per 100,000 [16]. Currently, the relationship between *BAP1* mutations and the prognosis of GBC is unknown, and no functional analysis of *BAP1* in GBC cell lines has yet been reported.

DNA methylation is known as one of the epigenetic mechanisms that controls and maintains gene expression without changing the DNA base sequence [17]. Methylation of the genome of BAP1 was analyzed in melanoma [18], malignant mesothelioma [5], and RCC [19], but almost no decrease in BAP1 expression due to methylation was found. There are also no reports that review the methylation of BAP1 in ICC or GBC.

In this study, we performed functional analysis in vitro and investigated the prognosis of GBC according to the BAP1 expression in clinical specimens to elucidate the clinical significance of *BAP1*. The mechanism of the down-regulation of BAP1 was also examined in terms of the genomic mutation and DNA methylation.

## Materials and methods

### Cell lines

Human gallbladder cancer (GBC) cell lines, G-415 [20] and OCUG-1 [21], were used in our study. G-415 was obtained from the Cell Resource Center for Biomedical Research, Tohoku University (Sendai, Japan). OCUG-1 was purchased from JCRB Cell Bank (Osaka, Japan). The cell lines were obtained directly from these institutions and were passaged in our laboratory for less than 6 months after receipt. The cell lines were cultured at 37°C in a 5% CO2 humidified incubator with RPMI-1640 medium (Sigma-Aldrich, St. Louis, MO, USA) plus 10% fetal bovine serum (FBS) (Biowest, Nuaillé, France) and 1% penicillin-streptomycin (Gibco by Life Technologies, Grand Island, NY, USA).

### Reverse Transcription (RT) PCR

RNA was extracted using RNeasy Mini Kit (QIAGEN, Hilden, Germany) and analyzed by Nanodrop (Thermo Scientific, Wilmington, DE, USA). RNA (0.5 μg) was transcribed into cDNA (complementary DNA) using PrimeScript High Fidelity RT-PCR Kit (Takara Bio, Kyoto, Japan) or PrimeScript RT reagent Kit (Takara Bio), according to the manufacturer’s instructions. Complementary DNA was amplified using optimal PCR conditions and the product was confirmed by electrophoresis on a 3% agarose gel. The sequences of the primer pairs used in this study are shown in S1 Table.

### Quantitative real-time PCR

Quantitative real-time PCR was performed using a Step One Plus Real-Time PCR system (Applied Biosystems, Foster City, CA, USA) with FAST SYBR Green Master Mix (Applied Biosystems). The relative quantification of mRNA within the samples was performed using the 2^−ΔΔCt^ method, and the results were normalized to the expression of GAPDH **(**glyceraldehyde 3-phosphate dehydrogenase) as an internal control in each sample. The sequences of the primer pairs were the same as those used in the RT PCR.

### Western blotting

Antibodies used for western blotting were as follows: as primary antibodies, mouse anti-human BAP1 monoclonal antibodies (sc-28383) were purchased from Santa Cruz Biotechnology (Santa Cruz, CA, USA), and rabbit monoclonal antibodies against human E-cadherin (#3195), vimentin (#5741) and GAPDH (#2118) were obtained from Cell Signaling Technology (Danvers, MA, USA); as secondary antibodies, anti-mouse IgG HRP-conjugated secondary antibody (#7076) and anti-rabbit IgG HRP-conjugated secondary antibody (#7074) were also purchased from Cell Signaling Technology.

To isolate total protein, cells were lysed in radio-immunoprecipitation assay (RIPA) buffer (Thermo Fisher Scientific, Waltham, MA, USA), consisting of 25 mM Tris–HCl [pH 7.6], 150 mM NaCl, 1% NP-40, 1% sodium deoxycholate and 0.1% sodium dodecyl sulfate (SDS). Protein densitometry was performed using Pierce BCA Protein Assay Reagent A and Reagent B (Thermo Scientific, Rockford, IL, USA) and NanoDrop2000 (Thermo Scientific). The cell lysate was diluted with 4x Laemmli sample buffer (Bio-Rad, Hercules, CA, USA) and NuPAGE sample reducing agent (Invitrogen, Carlsbad, CA, USA). Equal amounts of protein sample were applied on polyacrylamide gels (Mini-PROTEAN TGX Precast Gels) (Bio-Rad), separated by electrophoresis and electroblotted onto polyvinylidene difluoride (PVDF) membranes (Trans-Blot Turbo Transfer Pack) (Bio-Rad). After blocking with SuperBlock blocking buffer in tris buffered saline (TBS) (Thermo Scientific) and washing with TBS containing Tween 20 (TBS-T), the membranes were incubated overnight with primary antibodies at 4°C and then incubated with IgG HRP-conjugated secondary antibodies for 60 minutes at room temperature. Signals were detected using the Clarity Western ECL Substrate (Bio-Rad). Protein bands were visualized using an ImageQuant LAS 4000 mini system (GE Healthcare, Buckinghamshire, England, UK).

### siRNA transfection

BAP1 was knocked-down using specific small interfering RNA (siRNA) oligonucleotides (ON-TARGET plus Human BAP1 siRNA; Dharmacon, Lafayette, CO, USA). ON-TARGET plus Control Pool Non-Targeting Pool (Dharmacon) were used as the negative control. For transfection, the cationic lipid-mediated transfection method, using Lipofectamine RNAiMAX Reagent (Invitrogen), was adopted according to the manufacturer’s instructions. The sequences of the siRNA used in this study are shown in S2 Table.

### MTS assay

MTS assay using CellTiter 96 Aqueous One Solution Reagent (Promega, Madison, WI, USA), containing a tetrazolium compound [(3-(4, 5-dimethylthiazol-2-yl)-5-(3-carboxymethoxyphenyl)-2-(4-sulfophenyl)-2H-tetrazolium, inner salt; MTS] and an electron coupling reagent (phenazine ethosulfate; PES) was adopted as a colorimetric method for determining the number of viable cells in the cell proliferation or cytotoxicity assays. The absorbance at 490nm was recorded using a Multiskan FC Microplate Photometer (Thermo Scientific).

For the proliferation assay, BAP1 knocked-down GBC cells were seeded on 96-well plates and measured with the MTS assay on days 0, 1, 3, 5, and 7. The data were presented as relative increases of the average intensity compared to the control group.

For drug sensitivity, BAP1 knocked-down cells were incubated on 96-well plates for 3 days after administration of the drugs, gemcitabine (GEM; Wako, Osaka, Japan), fluorouracil (5-FU; Wako), cisplatin (CDDP; Wako), sodium valproate (Wako), 5-azacytidine (Wako), and bortezomib (Wako). Cell viability was measured by MTS assay and the obtained data were analyzed by calculating the area under the curve (AUC) of each group using GraphPad Prism (ver.7.0, GraphPad Software, San Diego, CA, USA).

### Scratch assay

Migration assays were performed using a scratch assay. A “scratch” was created with a 1–200 μL pipet tip on a monolayer of confluent cells cultured on 6-well cell plates. The images of the scratches were captured at the beginning and at 24 hours after incubation with RPMI-1640 medium not containing fetal bovine serum (FBS) during the cell migration. Digital images of the gap closure were obtained under a microscope (BZ-9000, KEYENCE, Tokyo, Japan). The migration area was analyzed using ImageJ software [22], and the data were presented as the relative increase in average migration area compared to the control group.

### Invasion assay

Invasion assays were performed using a Corning BioCoat Matrigel Invasion Chamber (Corning, NY, USA) according to the manufacturer’s instructions. The BAP1 knocked-down GBC cells that invaded through the pores to the lower surface of the filters after 20 hours were fixed and stained using 1% crystal violet (Wako, Osaka, Japan). The cell invasion was defined as the percentage of cell density at 20 hours compared to that seeded at 0 hours in five selected microscope fields. The data were presented as the relative increase of the average cell density to the control group.

### Human samples

Human samples were obtained from 10% formalin-fixed paraffin-embedded (FFPE) specimens, which were surgically resected from patients who received surgery under a diagnosis of gallbladder cancer (n = 47) at the Department of Surgery in Tohoku University Hospital between January 2005 and December 2016. Tissue specimens were encoded to protect patient confidentiality and processed under protocols approved by the Ethics Committee of Tohoku University Graduate School of Medicine.

### Immunohistochemistry

The Histofine Kit (Nichirei Biosciences, Tokyo, Japan), which uses the streptavidin–biotin amplification method, was adopted for immunohistochemical analysis. Mouse monoclonal antibodies targeting human BAP1 (Santa Cruz, CA, USA) were used for immunohistochemistry. The antigen–antibody complex was visualized with 3, 3′-diaminobenzidine solution consisting of 3, 3′-diaminobenzidine, Tris–HCl buffer, and H_2_O_2_. The sections were then counterstained with hematoxylin, dehydrated in a graded series of alcohol, permeated in xylene, mounted and observed under a microscope (BZ-9000).

Immunostained sections were analyzed using a HistoFAXS image cytometer (TissueGnostics, Vienna, Austria) and the specific density of diaminobenzidine in the gallbladder cancer cells was compared to that in hepatocytes using HistoQuest software (TissueGnostics). The density of diaminobenzidine in the gallbladder cancer cell samples not containing liver tissue in the specimen (N = 14) was compared to the median value of that in hepatocytes of the other 33 samples containing liver tissue in the specimen.

### DNA sequencing analysis

Genomic DNA was extracted from manually-collected samples of the cancer tissue portion in the FFPE tissue specimens using the QIamp DNA mini kit (QIAGEN) according to the FFPE protocols. DNA yield and quality were determined using Nanodrop (Thermo Scientific). For sequencing the 17 coding exons of *BAP1*, the primers listed in S4 Table were designed using Primer-BLAST (National Center for Biotechnology Information, Bethesda, MD, USA). PCR amplification was performed in a total volume of 25μl containing 100ng DNA, 10pmol of each primer and 0.625 units of PrimeSTAR HS Premix (Takara Bio). DNA amplification was performed in a PCR Thermal cycler Dice (Takara Bio). The PCR was started with 10 seconds at 98°C followed by 40 cycles of denaturation for 10 seconds at 98°C, annealing at 60°C for 15 seconds and extension at 72°C for 90 seconds followed by a final extension at 72°C for 90 seconds and cooling down for 10 minutes at 4°C. All PCR products were purified with QIAquick PCR Purification Kit (QIAGEN).

Capillary Sanger sequencing was conducted using an Applied Biosystems 3730xl DNA analyzer (Applied Biosystems) in Macrogen Japan Corp. (Kyoto, Japan)

### Methylation analysis

Methylation-specific PCR (MSP) was adopted for methylation analysis of the CpG island of *BAP1*. The UCSC Genome Browser (http://genome.ucsc.edu/cgi-bin/hgGateway) was used for searching the CpG island of *BAP1*, and MethPrimer (http://www.urogene.org/methprimer/index1.html) was utilized for the methylated-specific primer (M primer) and unmethylated-specific primer (UM primer). Sequences of the M primer and UM primer sets are shown in S3 Table.

Bisulfite modification of 2μg of genomic tumor DNA was performed using EpiTect Fast Bisulfite Conversion Kits (QIAGEN) following the manufacturer’s instructions. Approximately 100ng of bisulfite-modified DNA were used as a template for PCR amplification with the M primer and UM primer. The PCR product was confirmed by electrophoresis on a 3% agarose gel. For the control of methylated and unmethylated DNA, EpiTect PCR Control DNA Set (QIAGEN) was used.

### Statistical analysis

Quantitative data (mean ± SEM) were analyzed using Student’s *t*-test, Fisher’s exact test, one-way ANOVA, two-way ANOVA, and Mann–Whitney *U* test. Drug sensitivity was compared with the AUC of each group. Overall survival was calculated by Kaplan–Meier analysis and compared by log-rank tests. The Cox proportional hazards model was used to examine prognostic factors. Differences were considered significant when *p* < 0.05.

## Results

### BAP1 expression in GBC cell lines

BAP1 expression in the GBC cell lines G-415 and OCUG-1 was evaluated by RT-PCR and western blotting. In RT-PCR, the band corresponding to BAP1 was recognized in G-415 but hardly confirmed in OCUG-1 (S1 Fig). This indicated the expression of *BAP1* mRNA in G-415 and the down-regulation in OCUG-1. Western blotting showed the same result as RT-PCR. The band of BAP1 was observed strongly in G-415 but hardly recognized in OCUG-1 (S2 Fig). From the above results, BAP1 is considered to be expressed strongly in G-415 but scarcely at all in OCUG-1. The BAP1 positive GBC cell line G-415 was used for further analysis using siRNA for BAP1.

### Down-regulation of BAP1 in GBC cell line by siRNA

The GBC cell line G-415 was transfected with siRNA against BAP1, and western blotting using anti-BAP1 antibody was performed (S3A Fig). The expression of BAP1 was measured as the average luminance of the target band by ImageJ and compared with the control (luminance: 113.8). Suppression of BAP1 expression was observed in siRNA1 (luminance: 51.06), siRNA2 (luminance: 56.73), and siRNA3 (luminance: 67.87) (S3B Fig). siRNA1 and siRNA2, which have strong inhibitory effects on expression, were used for functional analysis of the proliferation, migration, and invasion.

### Proliferation was not affected by BAP1 down-regulation

**Fig 1:**
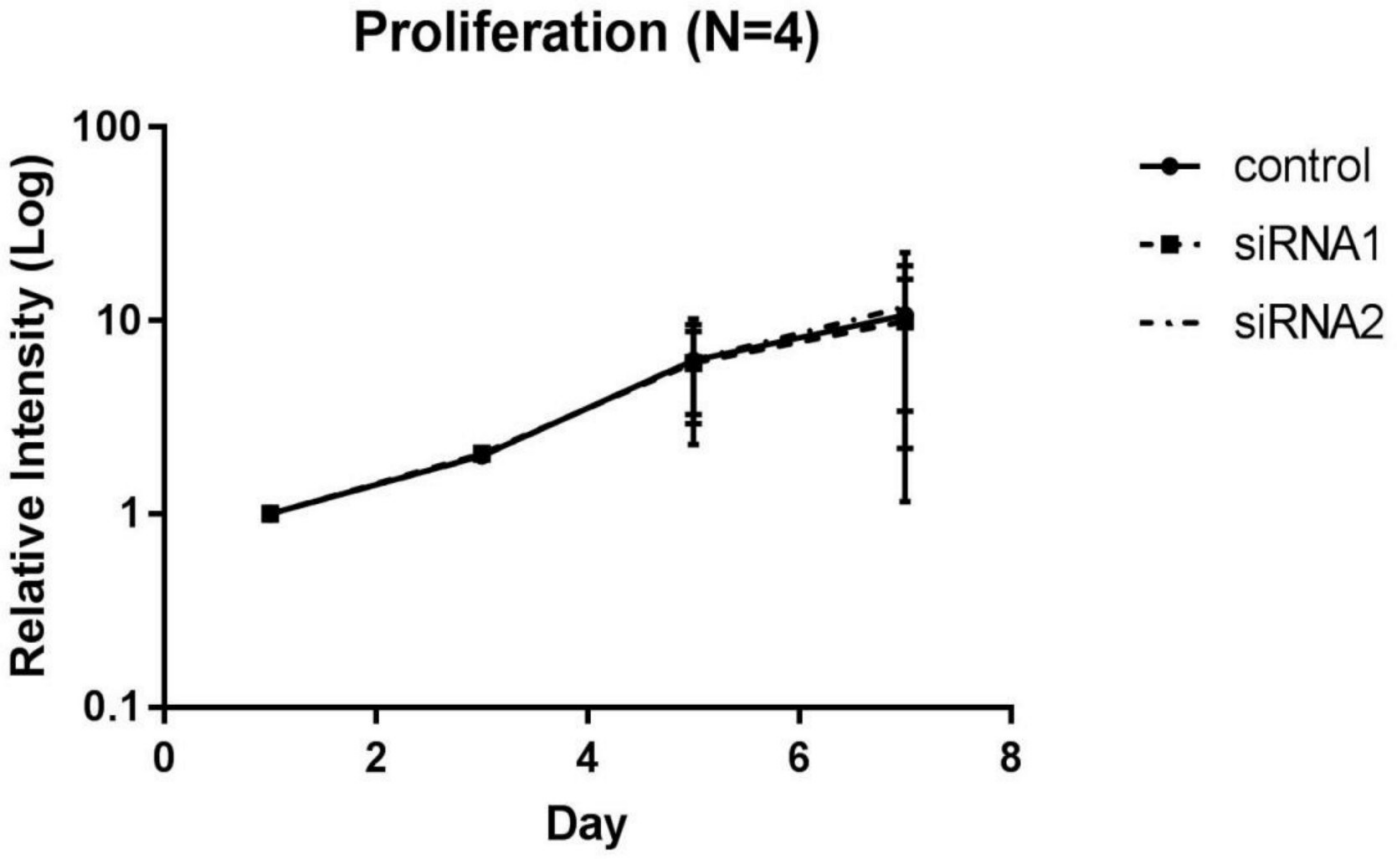
Proliferation assay in BAP1 knockdown GBC cell line. BAP1 expression of the GBC cell line G-415 was down-regulated by siRNA and the proliferative ability was measured by MTS assay. No significant difference was observed between each siRNA group and the control. The vertical axis shows the relative intensity compared to the intensity at day 1, and the horizontal axis shows the number of cell culture days.

To investigate the change in cell proliferation by the down-regulation of BAP1, MTS assay was performed on BAP1 knocked-down G-415 cells. The proliferative ability on day 1, day 3, day 5, and day 7 was 1, 2.00 ± 0.02, 6.22 ± 3.29, and 10.68 ± 8.50 fold, respectively, in the control group; 1, 2.05 ± 0.15, 6.03 ± 2.76, 9.90 ± 6.50 fold, respectively, in the siRNA1 group; 1, 1.99 ± 0.02, 6.24 ± 3.96, and 11.8 ± 10.65 fold, respectively, in the siRNA 2 group (control vs siRNA1; p = 0.99, control vs siRNA2; p = 0.99) (Fig 1). From the above results, it was considered that suppression of the BAP1 expression did not significantly affect the proliferation ability in the GBC cell line.

### Migration was promoted by BAP1 down-regulation

**Fig 2:**
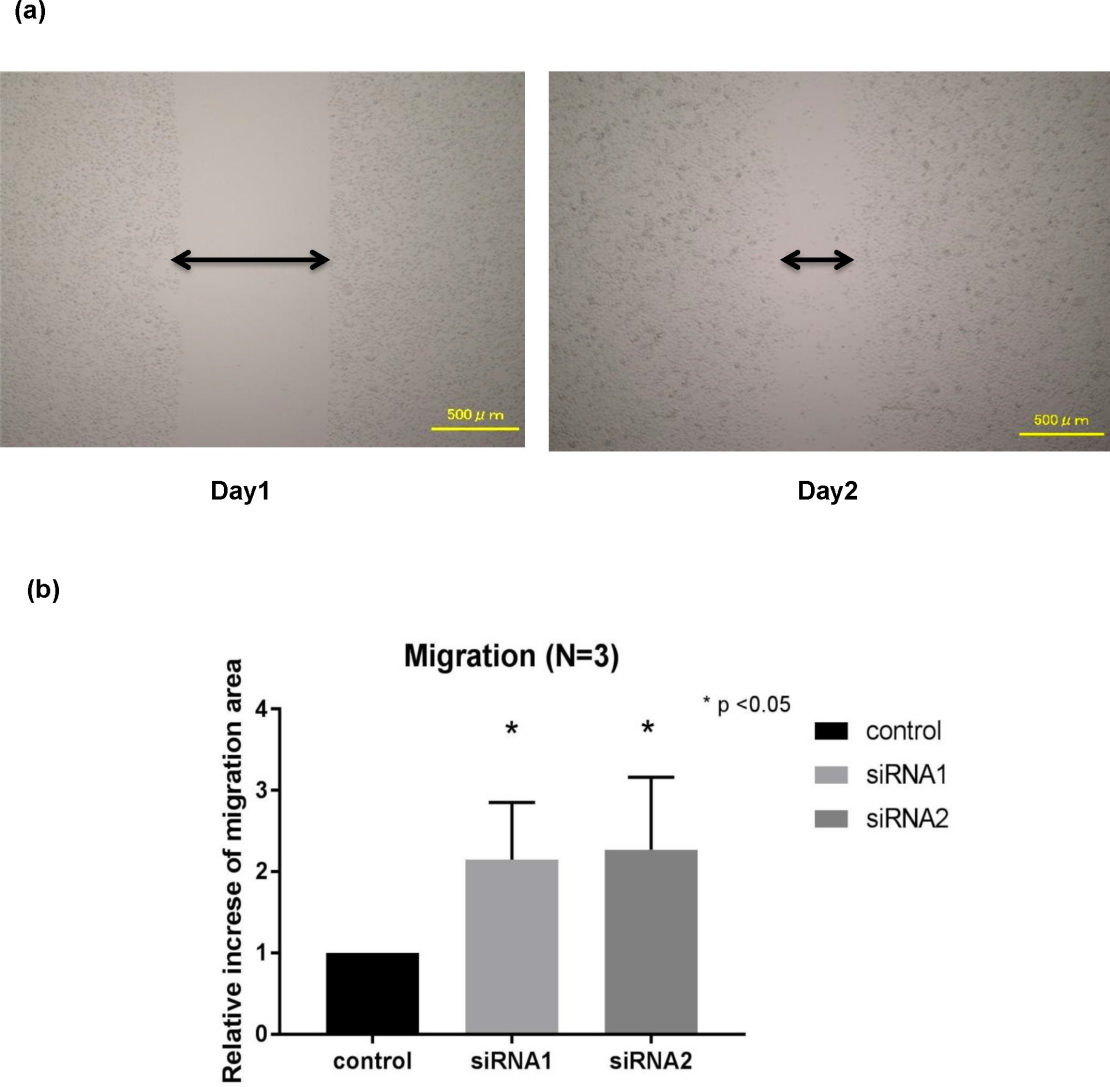
Migration assay in BAP1 knockdown GBC cell line. (a) A diagram immediately after scratching in the confluently cultured cells (day 1) and the narrowed gap by migrating cells (day 2) are shown. (b) The relative increase of the migration area compared to the control group after 24 hours was significantly higher in the BAP1 knocked-down cells.

To investigate the migratory ability of the BAP1 down-regulated cells, scratch assay was performed on BAP1 knocked-down G-415 cells (Fig 2A). Compared to the control group, the scratch area significantly decreased by 2.14 ± 0.25 fold (p < 0.05) in the siRNA1 group and 2.27 ± 0.32 fold (p < 0.05) in the siRNA2 group (Fig 2B). The suppression of BAP1 expression was thought to enhance cell migration.

### Invasion was promoted by BAP1 down-regulation

**Fig 3:**
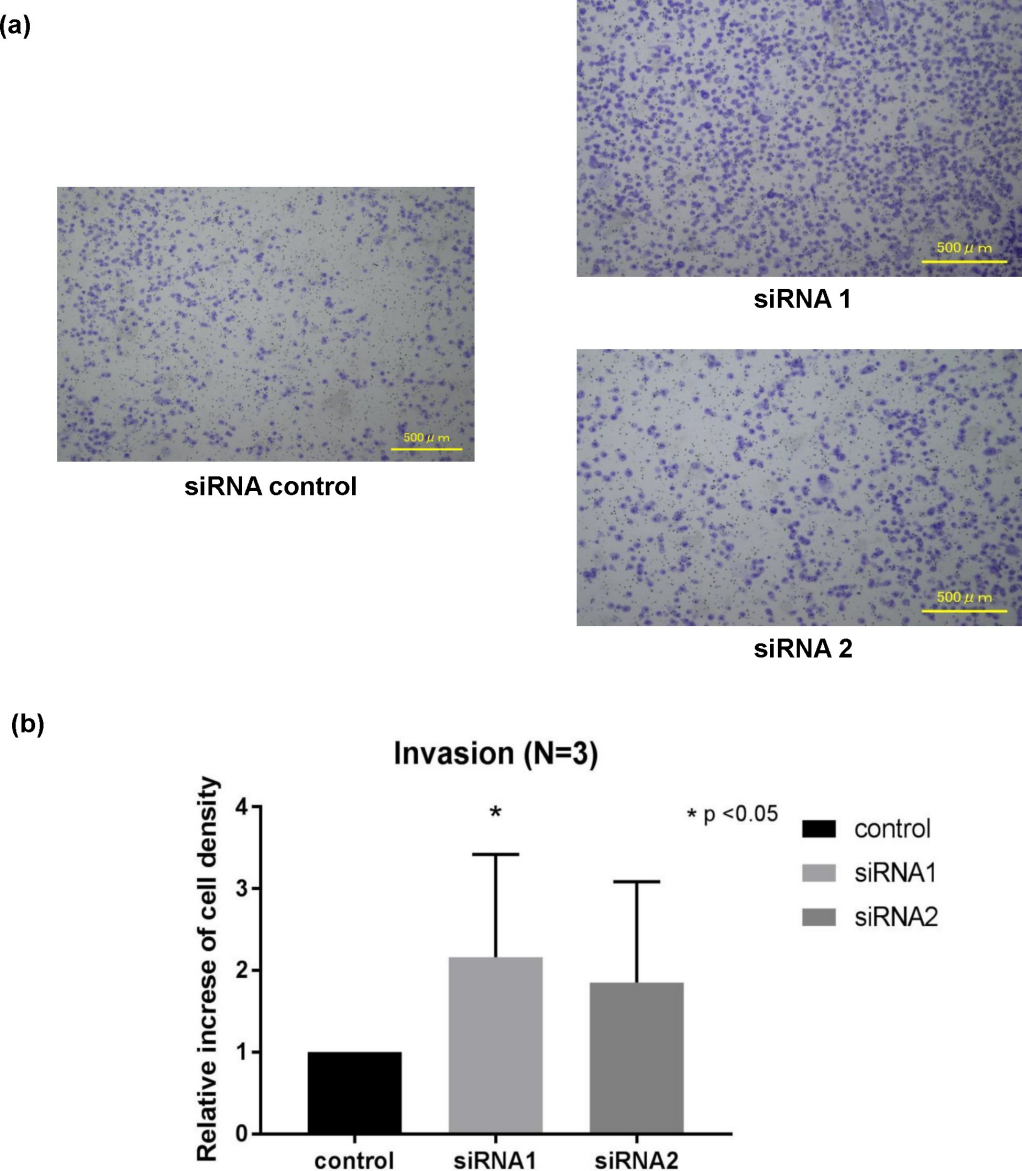
Invasion assay in BAP1 knockdown GBC cell line. (a) The GBC cell line G-415 was transfected with siRNA against BAP1 and was seeded on a Matrigel invasion chamber and the infiltrated cells on the lower surface of the chamber were stained. (b) Compared to the control group, the number of infiltrated cells tended to increase in both siRNA1 and siRNA2, and the number of infiltrated cells was significantly higher in the siRNA1.

To investigate the invasive ability, invasion assay using a Matrigel invasion chamber was performed on BAP1 knocked-down G-415 cells (Fig 3A). Compared to the control group, the number of cells on the lower surface of the chamber increased to 2.16 ± 0.36 fold (p < 0.05) in the siRNA1, and 1.85 ± 0.36 fold in the siRNA2 (p = 0.08) (Fig 3B). Since proliferation was not affected by BAP1 down-regulation, the difference in the number of cells was mainly due to the cells that had passed through the chamber, which means the invasive ability was enhanced by suppressing the expression of BAP1.

### EMT was not involved in the elevated migration and invasion of BAP1 down-regulation

We suspected that the main mechanism of the enhanced ability of migration and invasion in BAP1 knocked-down GBC cells was epithelial mesenchymal transition (EMT). EMT was evaluated by examining the expression of E-cadherin as an epithelial marker and vimentin as a mesenchymal marker in western blotting. Compared to the control group, no difference was observed in the expression of E-cadherin or vimentin in BAP1 knocked-down G-415 cells (S4 Fig).

### Sensitivity to bortezomib was attenuated by BAP1 down-regulation

**Fig 4:**
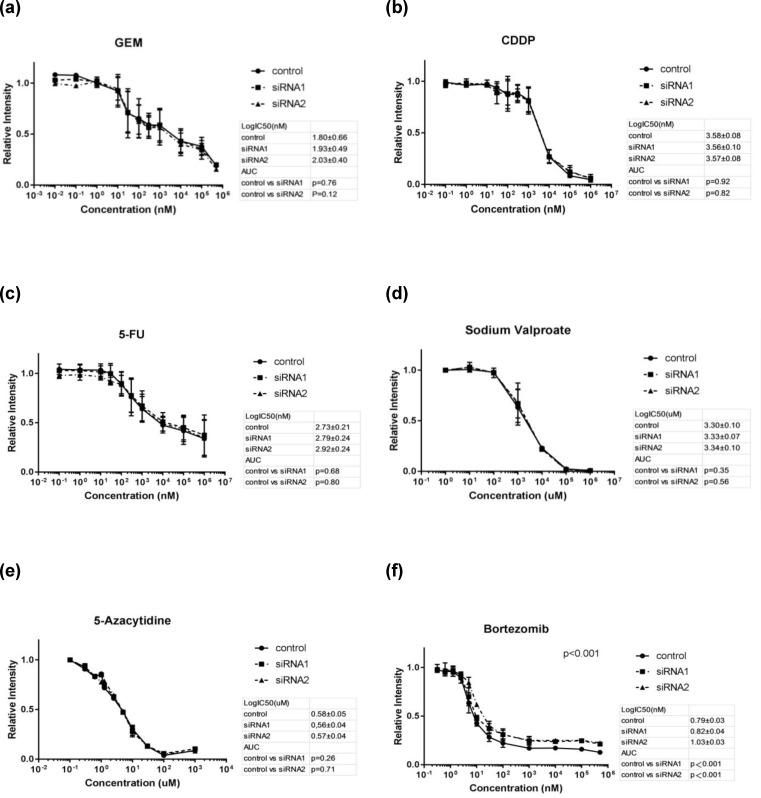
Drug sensitivity test in BAP1 knockdown GBC cell line. Cell viability of BAP1 knocked-down GBC cell line G-415 under the agents of gemcitabine (GEM), cisplatin (CDDP), fluorouracil (5-FU), sodium valproate, 5-azacytidine, and bortezomib was measured by MTS assay. No change in sensitivity was observed in GEM, CDDP, 5-FU, sodium valproate or 5-azacytidine. Bortezomib showed a significant decrease in sensitivity in the siRNA1 and siRNA2 group compared to the control.

**Fig 5:**
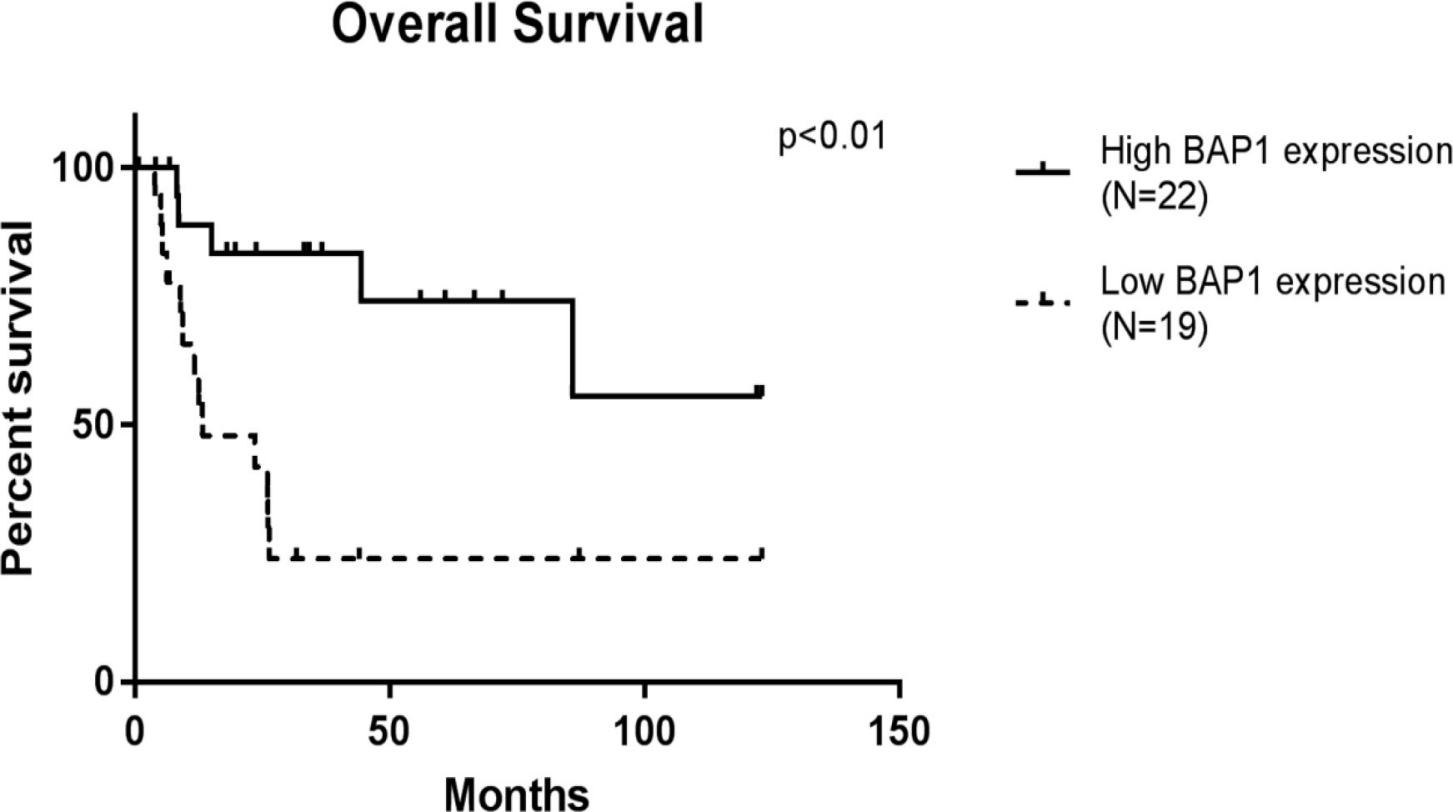
Kaplan–Meier survival analysis of BAP1 expression. Kaplan–Meier survival curves for 41 GBC cases except for in-hospital deaths and deaths from other diseases show a significantly poorer prognosis in the BAP1 low expression group.

The MTS assay was performed on BAP1 knocked-down G-415 cells to investigate whether the sensitivity to drugs was affected by the suppression of BAP1. No significant change was observed between BAP1 knocked-down cells and the control under the anticancer drugs for the GBCs: gemcitabine (control vs siRNA1; p = 0.76, control vs siRNA2; p = 0.12, Fig 4A), cisplatin (CDDP) (control vs siRNA1; p = 0.92, control vs siRNA2; p = 0.82, Fig 4B), and fluorouracil (5-FU) (control vs siRNA1; p = 0.68, control vs siRNA2; p = 0.80, Fig 4C). Sodium valproate, a histone deacetylase inhibitor [23], (control vs siRNA1; p = 0.35, control vs siRNA2; p = 0.56, Fig 4D) and 5-azacytidine, a DNA methyltransferase inhibitor [24], (control vs siRNA1; p = 0.26, control vs siRNA2; p = 0.71, Fig 4E) also had no significant effect on the sensitivity. A significant decrease in susceptibility to bortezomib, a proteosome inhibitor, was observed in BAP1 knocked-down cells compared with the control group (control vs siRNA1; p < 0.001, control vs siRNA2; p < 0.001, Fig 4F).

### Low expression of BAP1 was associated with poor survival in GBC

Immunohistochemistry of BAP1 in 47 cases of GBC revealed 25 cases with high BAP1 expression and 22 cases with low BAP1 expression (Table 1). The average age was 68 (46–86) years in the high BAP1 expression group (BAP1-H), 69.1 (46–83) years in the low BAP1 expression group (BAP1-L), and there was no significant difference (p = 0.71). There were 12 men and 13 women in the BAP1-H group, and 9 men and 13 women in the BAP1-L group, with no significant difference (p = 0.77). The stage classification of gallbladder cancer according to the UICC system [25] was 2:4:3:11:5 (stage 0:I:II:III:IV) in the BAP1-H group and 1:2:3:6:10 (stage 0:I:II:III:IV) in the BAP1-L group. The proportion of stage IV GBC was higher in the BAP1-L group compared to the BAP1-H group, but no significant difference was observed (p = 0.41). The R0 (no residual tumor) resection rate was 72% in the BAP1-H group and 50% in the BAP1-L group, with no significant difference (p = 0.14).

**Table 1:** Clinical characteristics of the 47 cases of GBC patients.

	High expression	Low expression	P value
Number of cases	25	22	
Age (range)	68.0 (46–86)	69.1 (46–83)	0.71
Sex (M 21:F 26)	12: 13	9: 13	0.77
Stage (0 : I : II : III : IV)	2 : 4 : 3 : 11 : 5	1 : 2 : 3 : 6 : 10	0.41
R0 surgery	72% (18/25)	50% (11/22)	0.14

A Kaplan–Meier survival curve was generated from the data of 41 patients, excluding in-hospital deaths and deaths from other diseases, and the prognosis was significantly poorer for the BAP1-L group (p < 0.01, Fig 5) (50% survival time was 13.3 months in the BAP1-L group, and it could not be calculated in the BAP1-H group because it was 50% or more). The survival analysis for 28 cases of advanced stage III and IV showed a similar result of significantly poorer prognosis in the BAP1-L group (p < 0.01, S5 Fig). (The 50% survival time was 11.7 months for the BAP1-L group and 85.8 months for the BAP1-H group). Multivariate analysis was performed with the primary tumor site (T), the regional lymph node involvement (N), the presence or otherwise of distant metastatic spread (M), the extent of residual disease (R), differentiation, infiltration, histological type, and BAP1 expression by the Cox proportional hazards model; N (p < 0.001), M (p < 0.01), R (p < 0.001), and BAP1 expression (P < 0.05) were detected as significant prognostic factors.

### DNA sequencing detected a number of genomic mutations in *BAP1* in GBC

**Fig 6:**
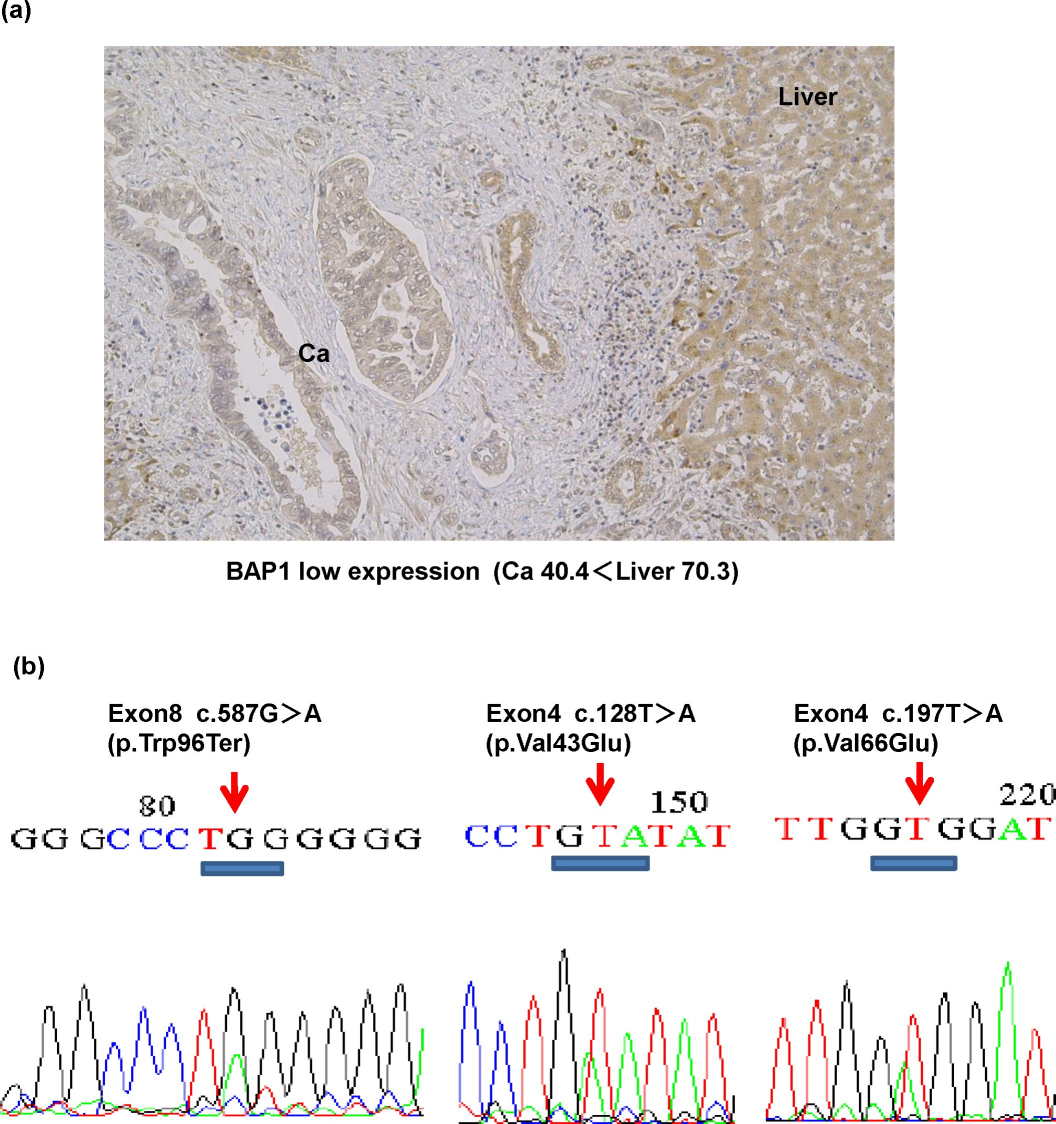
BAP1 expression and detected mutations in clinical GBC (ID 12). (a) Since the BAP1 staining concentration was low in the cancer part (Ca 40.4 < Liver 70.3), ID12 was categorized to the BAP1 low expression group. (b) The nonsense mutation on exon 8 (c.587G>A (p.Trp96Ter)) and the missense mutation on exon 4 (c.128T>A (p.Val43Glu), c.197T>A (p.Val66Glu)) were observed.

The results of the sequencing of 17 exons of *BAP1* of the GBC cell lines and clinical specimens are shown in Table 2. Neither G-415 nor OCUG-1 showed genetic mutations of *BAP1*. In the clinical specimens, 13 of the 47 patients had genetic nonsynonymous mutations of *BAP1*, consisting of 2 out of 25 cases in the BAP1-H group and 11 out of 22 cases in the BAP1-L group. Representative examples of *BAP1* mutation and BAP1 expression are shown in Fig 6 and S6 Fig. Mutations are described according to the Human Genome Variation Society guidelines (http://www.hgvs.org/mutnomen/).

**Table 2:** Summary of clinical characteristics, BAP1 expression, mutation, homozygous deletion and methylation in the GBC patients.

ID	Age (year)	sex	St.	BAP1 Expression	BAP1 Mutation	Affected Exon	Description of Mutation	Homozygous Deletion (Suspected)	Methylation
ID1	60	F	3	High	No				UM
ID2	65	M	1	High	Yes	Exon9	c.705C>A (p.Pro235 = )		UM
						Exon17	c.2166C>A (p.Arg722 = )		
ID3	73	F	3	High	Yes	Exon8	c.651C>T (p.Ala217 = )		UM
ID4	71	M	3	High	No				PM
ID5	70	M	3	High	No				UM
ID6	72	M	4	High	Yes	Exon9	c.747G>A (p.Lys249 = )		PM
ID7	76	F	3	Low	No				UM
ID8	72	F	3	High	No				UM
ID9	72	F	3	High	No				UM
ID10	66	M	3	High	Yes	Exon8	c.591G>A (p.Gly197 = )		UM
ID11	66	F	4	Low	Yes	Exon4	c.163G>T (p.Glu55Ter)		UM
						Exon9	c.747G>A (p. Lys249 = )		
ID12	74	F	3	Low	Yes	Exon4	c.128T>A (p.Val43Glu) c.197T>A(p.Val66Glu)		UM
						Exon8	c.587G>A (p.Trp196Ter)		
ID13	66	M	4	Low	No				UM
ID14	62	F	3	Low	Yes	Exon8	c.616G>A (p.Ala206Thr)		UM
						Exon9	c.697_698delinsTA(p.Val233Ter)		
						Exon14	c.1729G>T (p.Glu577Ter)		
ID15	70	M	4	High	No				UM
ID16	66	F	4	High	Yes	Exon8	c.652A>T (p.Thr218Ser)		UM
						Exon12	c.1219G>T (p.Asp407Tyr)		
ID17	80	M	4	Low	Yes	Exon4	c.232A>T (p.Asn78Tyr) c.243C>A (p.Phe81Leu)		UM
						Exon11	c.985C>T (p.Pro329Ser) c.1021A>T (p.His341Leu)		
						Exon15	c.1903C>A (p. Leu 635Met)		
ID18	72	M	4	Low	Yes	Exon11	c.996C>T (p.Pro332 = )	Exon13,14,15	UM
ID19	56	M	4	Low	Yes	Exon11	c.1027C>A (p.Leu343Ile) c.1048C>A (p.Pro350Thr)		UM
ID20	67	M	1	High	No				UM
ID21	75	M	4	Low	No				UM
ID22	54	F	4	High	No				UM
ID23	83	F	2	Low	No				UM
ID24	76	F	3	Low	Yes	Exon9	c.733C>T(p.Leu245 = )		UM
						Exon11	c.1036G>T (p.Val346Phe)		
ID25	78	F	1	Low	No				PM
ID26	70	F	1	High	No				UM
ID27	66	F	3	Low	No			Exon1,2,3,4,5	UM
ID28	76	F	2	Low	Yes	Exon9	c.746_747delinsTA (p.Lys249Ile)		UM
ID29	70	F	4	Low	No				UM
ID30	77	F	3	High	No				UM
ID31	86	M	1	High	No				UM
ID32	73	M	4	Low	Yes	Exon1	c.4_5insC c.9_10GG>TA (p.Asn2ThrfsTer3)		UM
						Exon14	c.1831G>T (p.Glu611Ter)		
ID33	78	M	2	High	No				UM
ID34	53	F	3	High	No				UM
ID35	47	M	2	Low	Yes	Exon4	c.131A>T (p.Tyr44Phe)		UM
						Exon11	c.1002A>G (p.Leu334 = )		
ID36	74	F	0	High	No				UM
ID37	70	F	3	High	No				UM
ID38	49	M	0	High	Yes	Exon9	c.1012C>T(p.Pro338Ser) c.1020C>T(p.Gly340Gly) c.1024A>T (p.Ser342Cys)		UM
ID39	70	M	4	Low	No				PM
ID40	82	F	0	Low	Yes	Exon9	c.721T>A (p.Tyr241Asn) c.746_747delinsTA (p.Lys249Ile)		PM
ID41	68	M	2	High	No				UM
ID42	71	M	2	High	No				UM
ID43	46	F	3	Low	No				PM
ID44	61	F	1	Low	No				UM
ID45	82	F	3	High	Yes	Exon4	c.249C>T (p.Ala83 = )		UM
ID46	66	M	4	Low	Yes	Exon11	c.965A>T (p.Gln322Leu)	Exon13,14,15,16,17	UM
ID47	46	F	4	High	No				UM

St., Stage; UM, Unmethylated; PM, Partially methylated. Synonymous substitution is highlighted in yellow, and non-synonymous substitution is in orange.

The mutations found in the BAP1-H group were: ID2, c.705C>A (p.Pro235 = ), and c.2166C>A (p.Arg722 = ); ID3, c.651C>T (p.Ala217 = ); ID6, c.747G>A (p.Lys249 = ); ID10, c.591G>A (p.Gly197 = ); ID16, c.652A>T (p.Thr218Ser), and c.1219G>T (p.Asp407Tyr); ID38, c.1012C>T (p.Pro338Ser), c.1020C>T (p.Gly340Gly), and c.1024A>T (p.Ser342Cys); ID45, c.249C>T (p.Ala83 = ). Seven mutations in five cases (ID2, ID3, ID6, ID10, and ID45) in the BAP1-H group showed synonymous substitutions in which the encoded amino acid was not altered.

On the other hand, the mutations in the BAP1-L group were: ID11, c.163G>T (p.Glu55Ter), and c.747G>A (p. Lys249 = ); ID12, c.128T>A (p.Val43Glu), c.197T>A (p.Val66Glu), and c.587G>A (p.Trp196Ter); ID14, c.616G>A (p.Ala206Thr), c.697_698delinsTA (p.Val233Ter), and c.1729G>T (p.Glu577Ter); ID17, c.232A>T (p.Asn78Tyr), c.243C>A (p.Phe81Leu), c.985C>T (p.Pro329Ser), c.1021A>T (p.His341Leu) and c.1903C>A (p. Leu 635Met); ID18, c.996C>T (p.Pro332 = ); ID19, c.1027C>A (p.Leu343Ile), and c.1048C>A (p.Pro350Thr); ID24, c.733C>T (p.Leu245 = ) and c.1036G>T (p.Val346Phe); ID28, c.746_747delinsTA (p.Lys249Ile); ID32, c.4_5insC c.9_10GG>TA (p.Asn2ThrfsTer3) and c.1831G>T (p.Glu611Ter); ID35, c.131A>T (p.Tyr44Phe), and c.1002A>G (p.Leu334 = ); ID40, c.721T>A (p.Tyr241Asn), and c.746_747delinsTA (p.Lys249Ile); ID46, c.965A>T (p.Gln322Leu). Nonsynonymous substitutions and/or frame shifts were found in eleven cases, with one case showing a synonymous substitution. The genetic mutations affecting the protein expression of BAP1 were significantly higher in the BAP1-L group (11 cases, 50%) than in the BAP1-H group (2 cases, 8%) (p = 0.003). Synonymous substitution mutations found in three cases of the BAP1-H group and two cases of the BAP1-L group are reported in polymorphic databases (dbSNP http://www.ncbi.nlm.nih In .gov / projects / SNP /); these were thought to be single-nucleotide polymorphisms (SNPs).

In addition, in three cases of the BAP1-L group (ID 18, ID 27, ID 46), homozygous deletion was suspected due to the lack of PCR products in the consecutive exons (Table 2).

The summary of mutations and homozygous deletion in the 47 cases of GBC patients are shown in Table 3.

**Table 3:** Summary of mutations and homozygous deletion in the 47 cases of GBC patients.

	High expression (25 cases)	Low expression (22 cases)	P value
Synonymous substitution (variant)	5 (20%)	1 (4.5%)	0.19
Nonsynonymous substitution	2 (8%)	11 (50%)	0.003
Homozygous deletion (HD)	0	3 (13.6%)	0.09
Nonsynonymous substitution and/or HD	2 (8%)	13 (59%)	0.0003

### Partial methylation of CpG island of BAP1 was detected in GBC

To investigate whether methylation is involved in the suppression of BAP1, DNA methylation analysis of the CpG island was performed in GBC cell lines and 47 GBC specimens (Table 2).

In the clinical specimens of GBC, 41 of 47 cases did not show bands for the M product (S7A Fig) and the other six showed weak bands for the M product (ID4, ID6, ID25, ID39, ID40, ID43) (S7B Fig), suggesting that partial methylation had occurred. Among six cases, two cases (ID4, ID6) were in the BAP1-H group and four cases (ID25, ID39, ID40, ID43) were in the BAP1-L group. There was no significant relationship between methylation in the GBC specimens and BAP1 expression (p = 0.40) or prognosis. (P = 0.71).

OCUG-1 showed strong bands, not only for the unmethylation-specific product (UM product), but also for the methylation-specific product (M product), while little M product was detected in G-415 (S7C Fig). To investigate whether methylation is involved in the regulation of BAP1 expression in OCUG-1, BAP1 expression was measured by quantitative real-time PCR with demethylation by 5-azacytidine (5-AZA). The expression level of BAP1 in OCUG-1 after demethylation was 1.83 ± 0.35 fold higher than that in the control (S8 Fig). The suppressed expression of BAP1 in OCUG-1 was therefore thought to be due to methylation.

## Discussion

The functional role of BAP1 down-regulation in cancer cells remains controversial. In a study of UM cell lines, the suppression of BAP1 by siRNA did not affect proliferation, migration or invasion [26]. Meanwhile, the suppression of BAP1 by siRNA in bile duct cancer cell lines enhanced the proliferative capacity [27]. In our study by knocking-down the expression of BAP1 by siRNA, the cell proliferation ability did not change, while the migratory and invasive abilities were enhanced, though we use one kind of GBC cell line. This result supports our clinical outcomes of more advanced stages of GBC and the poor prognosis for patients who show low BAP1 expression.

In order to investigate the mechanism of the enhanced ability of migration and invasion by BAP1 down-regulation, we analyzed EMT in BAP1 knocked-down cells, but failed to prove the positive involvement of EMT in which epithelial cells lost cell polarity and cell adhesion function with the surrounding cells and acquired mesenchymal properties [28]. As for the mechanism of invasion and migration, Onken *et al*. have reported that the down-regulation of BAP1 increases the transmigration of cancer cells, but does not affect intercalation in uveal melanoma UM cells [29]. Further in vitro studies are needed to elucidate the functional role of BAP1 down-regulation in cancer cells.

Although BAP1 down-regulation did not affect the drug sensitivity of antitumor agents used to treat GBC, we showed that BAP1 down-regulation attenuated the sensitivity to bortezomib, which is clinically applied to multiple myeloma and an inhibitor of proteasome that regulates the accumulation of the abnormal protein and lethal stress in cells [30]. The mechanism involved in the acquisition of tolerance to bortezomib has not been clarified, but the involvement of mutations of proteasome and chaperones in the endoplasmic reticulum has been reported in multiple myeloma [31]. Although bortezomib did not show a prognostic improvement in a phase II clinical trials for advanced cholangiocarcinoma [32], there is a report of the antitumor effects of bortezomib [33]. By selecting patients according to BAP1 expression, bortezomib could be a candidate for chemotherapy to treat GBC.

The frequency of mutation affecting protein expression in GBC detected in our study (8% in the BAP1-H group, 50% in the BAP1-L group, 27.6% in total) is higher than in previous reports (13% [8], 3.8% [9]). The detected mutations tended to be more common in the first half of BAP1, which contains domains such as UCH domain and BRCA 1 associated RING domain 1 (BARD 1) binding domain [1]. UCH is involved in the de-ubiquitin activity of BAP1 [2], and BAP1 interacts with BARD1 to inhibit the E3 ligase activity of BRCA1-BARD1 complex. BAP1 and BRCA1-BARD1 complex coordinately regulate ubiquitination during the DNA damage response and the cell cycle [34, 35]. Accordingly, it is speculated that mutations involved particularly in these regions greatly affect the decrease in the functional expression of BAP1.

FFPE samples show frequent sequence changes due to DNA damage resulting from formalin fixation and storage, most commonly manifesting as a cytosine to thymine transition caused by the deamination of cytosine (artifactual C>T and G>A transitions) [36]. We did not use this method to remove artificial transitions, but even if we exclude this type of mutation, the frequency of detected mutations of GBC in our study is still higher than that reported in the past. Our study showed significant involvement of BAP1 in GBC.

In addition to the genetic mutations found by DNA sequencing, homozygous deletion was suspected in three cases (ID 18, ID 27, ID 46) in which PCR products were not detected in consecutive exons. In BAP1 low expression mesothelioma, homozygous deletion was recognized in 76% of samples with fluorescence *in situ* hybridization (FISH) [37], so there might be a considerable number of deletions that we could not detect by DNA sequencing.

As for methylation, it might suppress the expression of BAP1 in OCUG-1 but, in clinical samples, methylation was occasional and weak and did not correlate with the BAP1 expression or prognosis of GBC. From the above, it seems that DNA methyltransferase inhibitors are less likely to be useful for BAP1, as reported in other cancers [5, 18, 19].

Since the point mutation in the exon region is mainly studied in our study of BAP1 expression, the mutation in the intron region and other epigenetic mechanism such as histone acetylation and post-translational modification were not taken into consideration. Although we could not demonstrate that the cause of low BAP1 expression was comprehensively analyzed, our study has revealed that genomic mutations of *BAP1* are strongly related to the suppression of BAP1 expression in GBC.

## Conclusion

Our study suggests that the suppressed expression of BAP1 mainly due to genetic mutations promotes the migratory and invasive ability of GBC cells and consequently correlates with a poor prognosis. BAP1 is a tumor suppressor gene in GBC that affects the prognosis and could be a target for therapy.

## Supporting information

S1 Table Sequences of primer of BAP1 and GAPDH for the RT-PCR. (PDF)

S2 Table Sequences of siRNA. (PDF)

S3 Table Sequences of M primer and UM primer. (PDF)

S4 Table Primer sequences used for 17 exon sequencing of DNA. (PDF)

S1 Fig Expression of BAP1 in the GBC cell lines by RT-PCR. (PDF)

S2 Fig Expression of BAP1 in the GBC cell lines by Western blotting. (PDF)

S3 Fig BAP1 down-regulation by siRNA in the GBC cell line. (PDF)

S4 Fig Epithelial mesenchymal transition (EMT) markers in the BAP1 knockdown GBC cell line. (PDF)

S5 Fig Kaplan–Meier survival analysis of BAP1 expression (Stage III, IV). (PDF)

S6 Fig BAP1 expression and detected mutations in clinical GBC (ID 14). (PDF)

S7 Fig DNA methylation analysis of CpG island of BAP1. (PDF)

S8 Fig BAP1 expression of BAP1-methylated GBC cell line after treatment with 5-azacytidine. (PDF)

S9 Fig Schema of domains and detected mutations of BAP1. (PDF)
