# Supplementary material for: Loss of BAP1 expression is associated with genetic mutation and can predict outcomes in gallbladder cancer
Source: PLoS One. 2018 Nov 5;13(11):e0206643. doi: 10.1371/journal.pone.0206643 (PMC6218052; doi:10.1371/journal.pone.0206643)
Supplement: S1 Table — (PDF) [file pone.0206643.s001.pdf]

S1 Table. Sequences of primer of BAP1 and GAPDH for the RT-PCR.

Primer	Sequences of primer
BAP1 (Forward)	5’-CCCCGCGGGAAGATGAATAA-3’
BAP1 (Reverse)	5’-ACCCCCTTGACACCGAAATC-3’
GAPDH (Forward)	5’-GCACCGTCAAGGCTGAGAAC-3’
GAPDH(Reverse)	5’-TGGTGAAGACGCCAGTGGA-3’
