# Supplementary material for: Loss of BAP1 expression is associated with genetic mutation and can predict outcomes in gallbladder cancer
Source: PLoS One. 2018 Nov 5;13(11):e0206643. doi: 10.1371/journal.pone.0206643 (PMC6218052; doi:10.1371/journal.pone.0206643)
Supplement: S2 Table — (PDF) [file pone.0206643.s002.pdf]

S2 Table. Sequences of siRNA.

siRNA	Sequences of siRNA
siRNA control	5′-UGGUUUACAUGUCGACUAAUU-3′<br>5′-UGGUUUACAUGUUGUGUGAUU-3′<br>5′-UGGUUUACAUGUUUUCUGAUU-3′<br>5′-UGGUUUACAUGUUUUCCUAUU-3′
siRNA1	5′- CAACCGUGCUGUCCGUGAU-3′
siRNA2	5′- CCAUCAACGUCUUGGCUGA-3′
siRNA3	5′- GAGCAAAGGAUAUGCGAUU-3′
