# Supplementary material for: Loss of BAP1 expression is associated with genetic mutation and can predict outcomes in gallbladder cancer
Source: PLoS One. 2018 Nov 5;13(11):e0206643. doi: 10.1371/journal.pone.0206643 (PMC6218052; doi:10.1371/journal.pone.0206643)
Supplement: S3 Table — (PDF) [file pone.0206643.s003.pdf]

S3 Table. Sequences of M primer and UM primer.

Primer	Sequences of primer
M-Forward	5’-AGTTATTTTTTTAGTTGTTTGAGGGC-3’
M-Reverse	5’-GTTAACGATCGATTTCTACTAACGAT-3’
UM-Forward	5’-GTTATTTTTTTAGTTGTTTGAGGGTGT-3’
UM-Reverse	5’-CCATTAACAATCAATTTCTACTAACAAT-3’
