# Supplementary material for: Loss of BAP1 expression is associated with genetic mutation and can predict outcomes in gallbladder cancer
Source: PLoS One. 2018 Nov 5;13(11):e0206643. doi: 10.1371/journal.pone.0206643 (PMC6218052; doi:10.1371/journal.pone.0206643)
Supplement: S4 Table — (PDF) [file pone.0206643.s004.pdf]

S4 Table. Primer sequences used for 17 exon sequencing of DNA.

Exon	Sequences of primer
Exon1/Exon2/Exon3 (Forward)	5’-CTGGGCCCGTTGTCTGTGT-3’
Exon1/Exon2/Exon3 (Reverse)	5’-ATCAGTTTGATCAGGAGCGG-3’
Exon4 (Forward)	5’-ACAAGGCCACTTCAGACACA-3’
Exon4 (Reverse)	5’-TTCAGTTCGTTCTGCCAGAGG-3’
Exon5 (Forward)	5’-TGAGTCCACTCTCTGTGTCCT-3’
Exon5 (Reverse)	5’-CCAATATCATGTGGTAGCATTCCC-3’
Exon6/Exon7 (Forward)	5’-CCACCCATAGTCCTACCTG-3’
Exon6/Exon7 (Reverse)	5’-TGCCACTGGGTACCACATAC-3’
Exon8 (Forward)	5’-GATTCCCTGGCTCAACTGCT-3’
Exon8 (Forward)	5’-GGCTCAACTGCTCTTCTCTGT-3’
Exon8 (Reverse)	5’-ACTCTCTGTCCCTCCCAAAGT-3’
Exon8 (Reverse)	5’-AGCCCAGGCAGGAAATAAGAC-3’
Exon9 (Forward)	5’-CTGCCAGGATATCTGCCTCAAC-3’
Exon9 (Reverse)	5’-AGCCCAGATCTACAAGAGAGT-3’
Exon10 (Forward)	5’-TGTGGGAAAGGTGGGACTTG-3’
Exon10 (Reverse)	5’-TCCCTCTACCTTCTGACGGG-3’

Exon11 (Forward)	5’-GGGGAGACTGTGAGCTTTTCT-3’
Exon11 (Reverse)	5’-AAAATTGCCTGTTGCAGCCTC-3’
Exon11 (Reverse)	5’-TGCCAGTAAAACCCAACCCAT-3’
Exon12 (Forward)	5’-GGCTTTCTCTCTGGCTGTGA-3’
Exon12 (Reverse)	5’-TCCGCAGGTGCTCAACATTA-3’
Exon13 (Forward)	5’-ACTGCTGGGTATGGTCACCT-3’
Exon13 (Reverse)	5’-TGCAGGACACTTTGTGGTCA-3’
Exon14 (Forward)	5’-GATCTGGGTCCTGTCATCAGC-3’
Exon14 (Forward)	5’-GCCAAGTGACCACAAAGTGTC-3’
Exon14 (Reverse)	5’-AATCAAGAACTTGGCACCTGG-3’
Exon14 (Reverse)	5’-CCAGCCACCAATCTTCACACC-3’
Exon15/Exon16 (Forward)	5’-TATTGCTCGTGGGGCTTTGT-3’
Exon15/Exon16 (Reverse)	5’-CAGGGGAGGGGAGCTGAA-3’
Exon17 (Forward)	5’-TTGAGCAGACCTTGGGGCAC-3’
Exon17 (Reverse)	5’-CTGGGAAAAGGGGAAGTGGG-3’
