# Supplementary figures and images for: Loss of BAP1 expression is associated with genetic mutation and can predict outcomes in gallbladder cancer
Source: PLoS One. 2018 Nov 5;13(11):e0206643. doi: 10.1371/journal.pone.0206643 (PMC6218052; doi:10.1371/journal.pone.0206643)

S1 Fig. Expression of BAP1 in the GBC cell lines by RT-PCR.

(a)

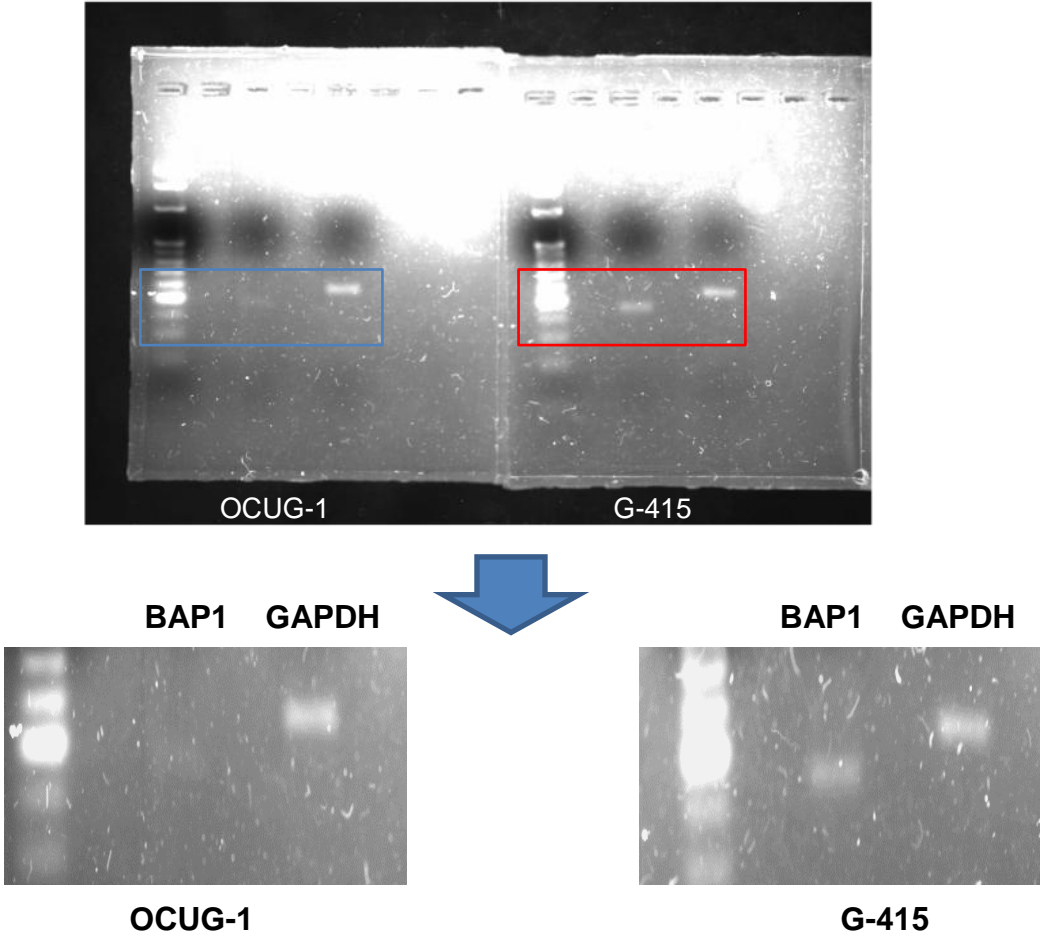

(b)

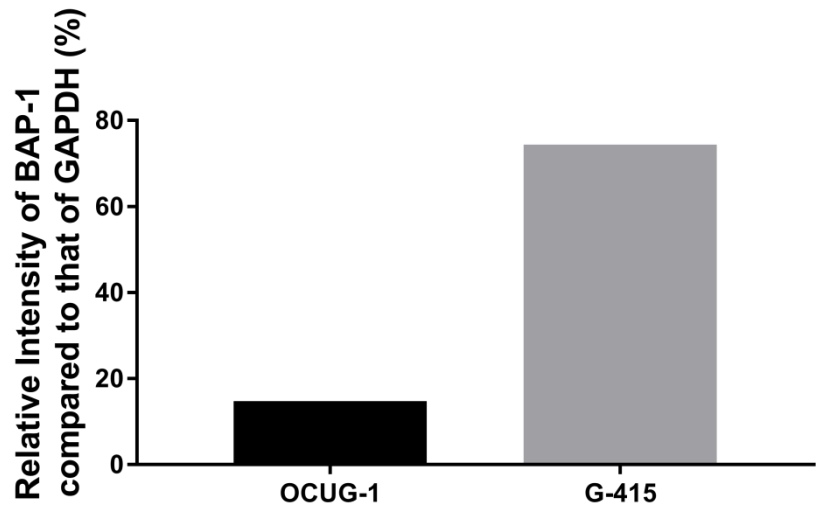

Supplement: S1 Fig — (PDF) [file pone.0206643.s005.pdf]

S2 Fig. Expression of BAP1 in the GBC cell lines by Western blotting.

(a)

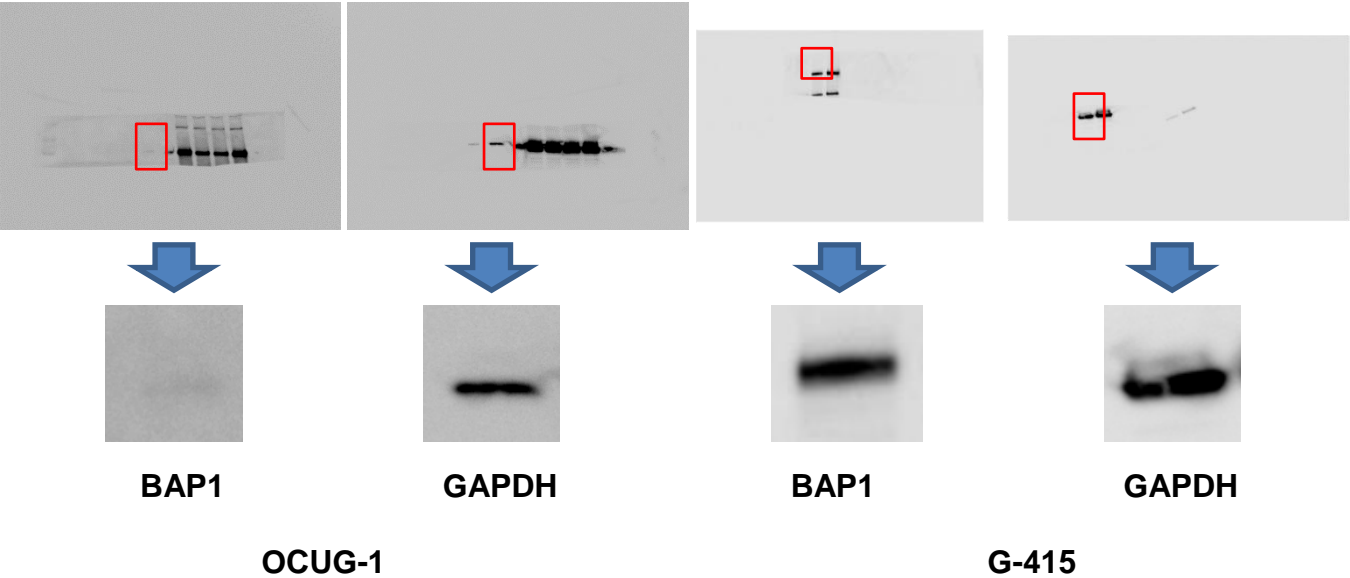

(b)

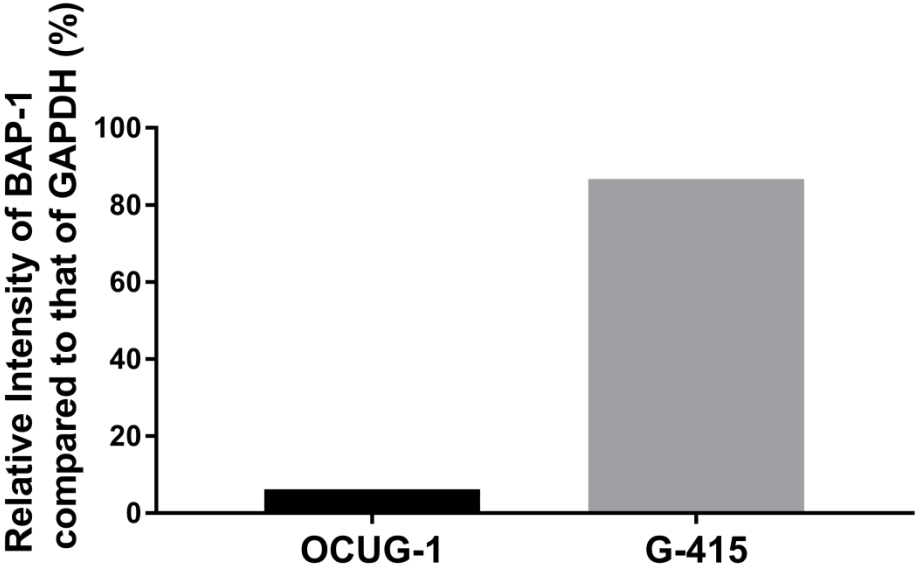

Supplement: S2 Fig — (PDF) [file pone.0206643.s006.pdf]

S3 Fig. BAP1 down-regulation by siRNA in the GBC cell line.

(a)

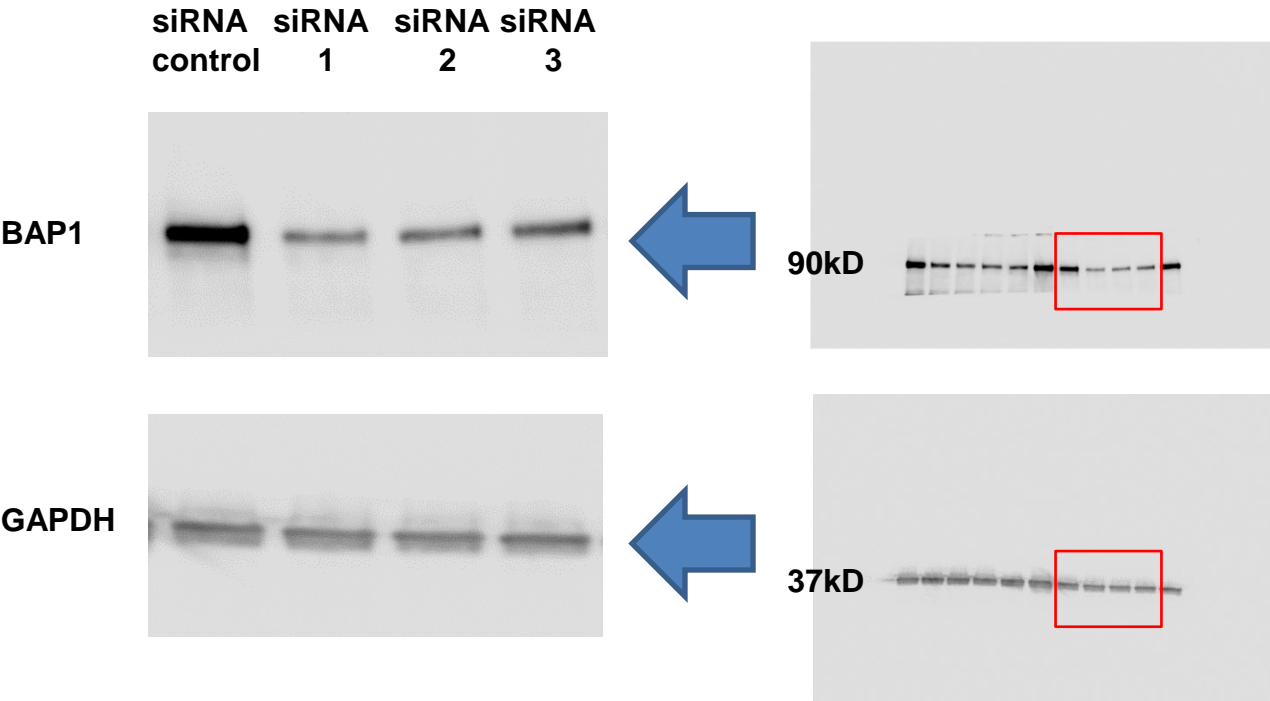

(b)

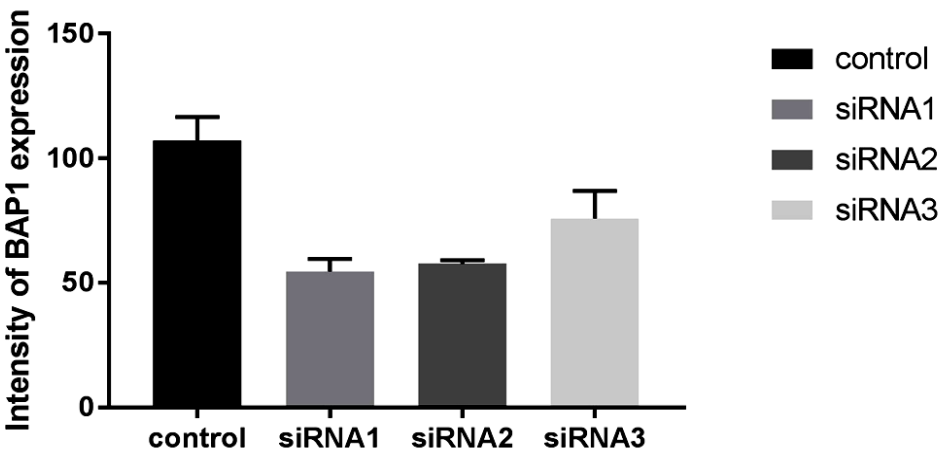

Supplement: S3 Fig — (PDF) [file pone.0206643.s007.pdf]

**S4 Fig. Epithelial mesenchymal transition (EMT) markers in the BAP1 knockdown GBC cell line.**

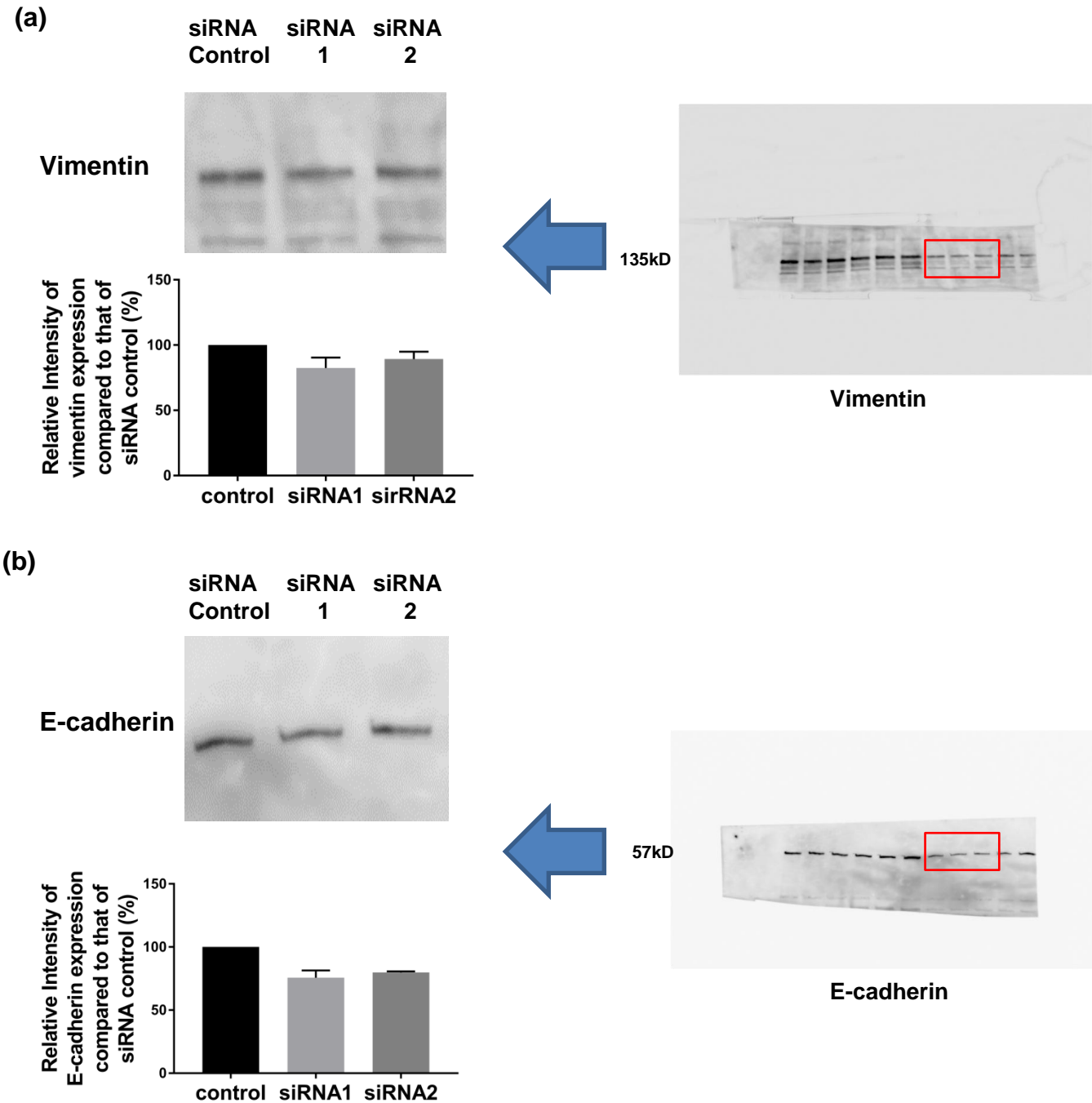

Supplement: S4 Fig — (PDF) [file pone.0206643.s008.pdf]

**S5 Fig. Kaplan–Meier survival analysis of BAP1 expression (Stage III, IV).**

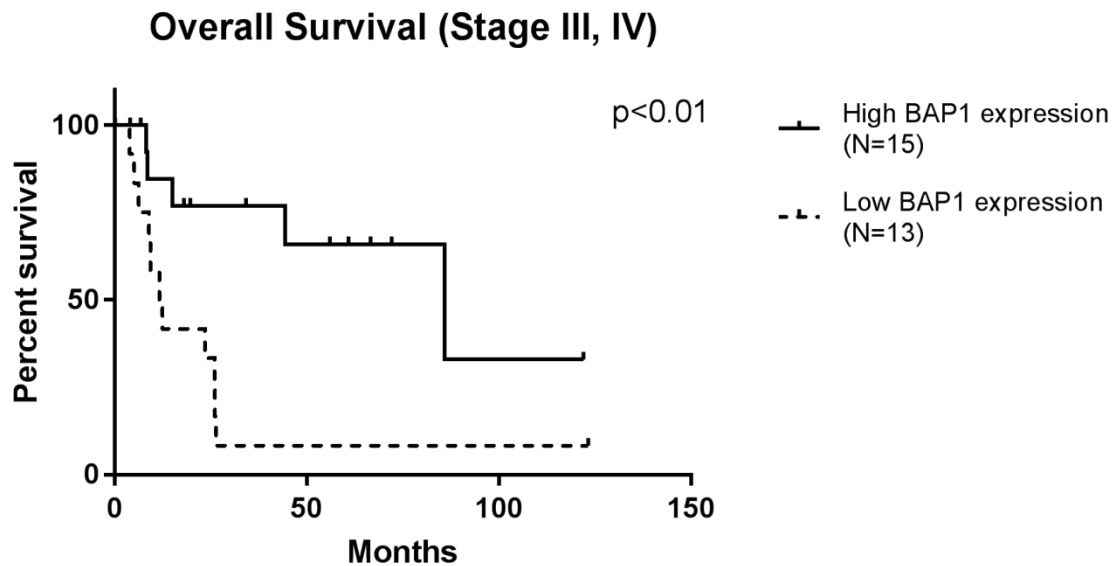

Supplement: S5 Fig — (PDF) [file pone.0206643.s009.pdf]

**S8 Fig. BAP1 expression of BAP1-methylated GBC cell line after treatment with 5-azacytidine.**

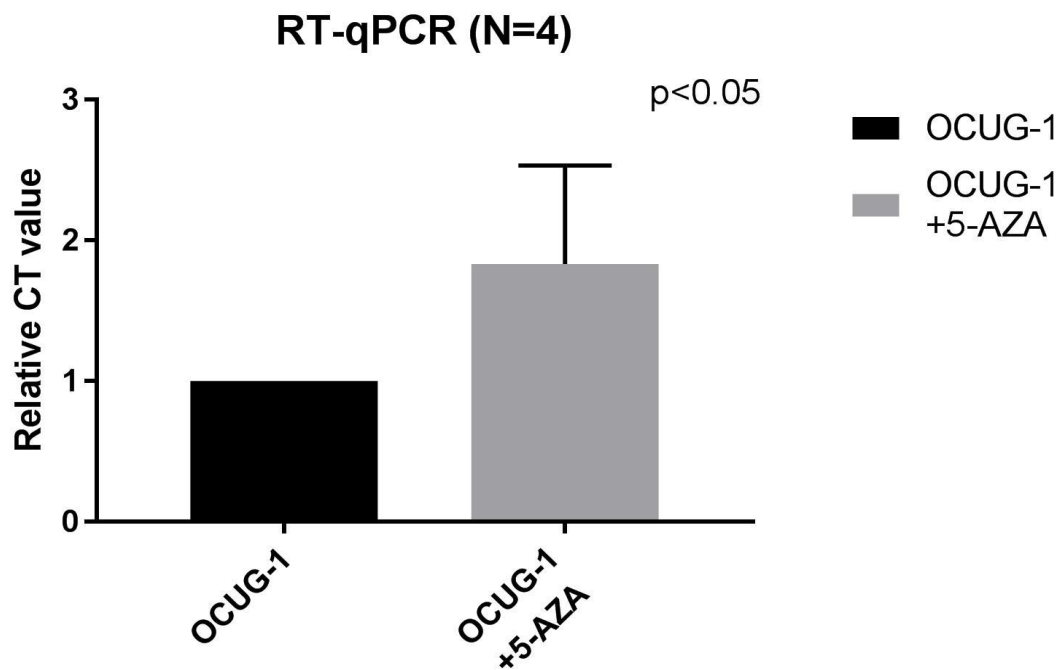

Supplement: S8 Fig — (PDF) [file pone.0206643.s012.pdf]
