# Supplementary material for: Loss of BAP1 expression is associated with genetic mutation and can predict outcomes in gallbladder cancer
Source: PLoS One. 2018 Nov 5;13(11):e0206643. doi: 10.1371/journal.pone.0206643 (PMC6218052; doi:10.1371/journal.pone.0206643)

S6 Fig. BAP1 expression and detected mutations in clinical GBC (ID 14).

(a)

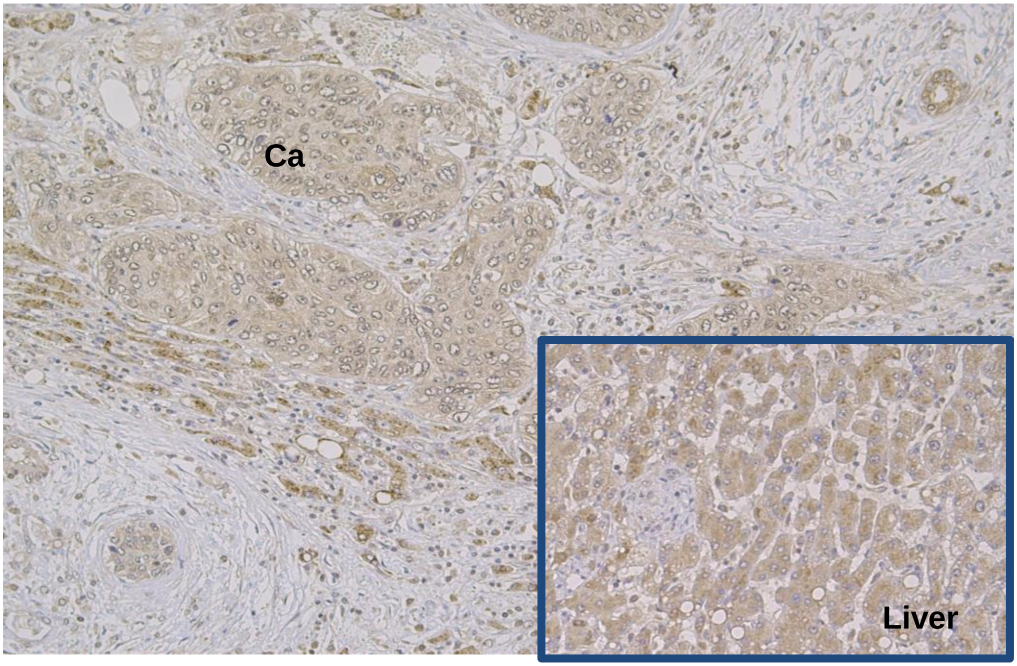

BAP1 low expression (Ca 47.9＜Liver 54.7)

(b)

Exon8 c.616G＞A
(p.Ala206Thr)

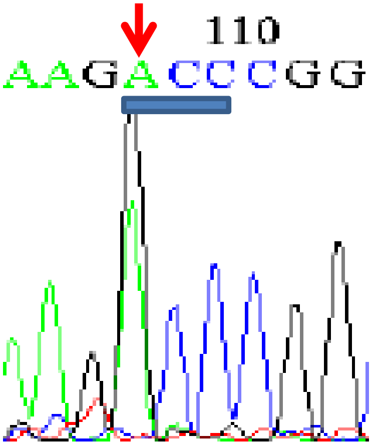

Exon9 c.697_698GT＞TA
(p.Val233Ter)

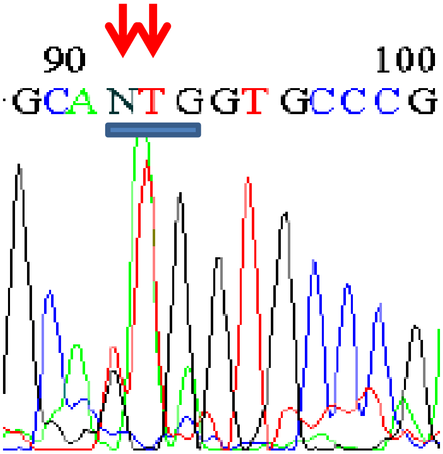

Supplement: S6 Fig — (PDF) [file pone.0206643.s010.pdf]
