# Supplementary material for: Loss of BAP1 expression is associated with genetic mutation and can predict outcomes in gallbladder cancer
Source: PLoS One. 2018 Nov 5;13(11):e0206643. doi: 10.1371/journal.pone.0206643 (PMC6218052; doi:10.1371/journal.pone.0206643)

S7 Fig. DNA methylation analysis of CpG island of BAP1.

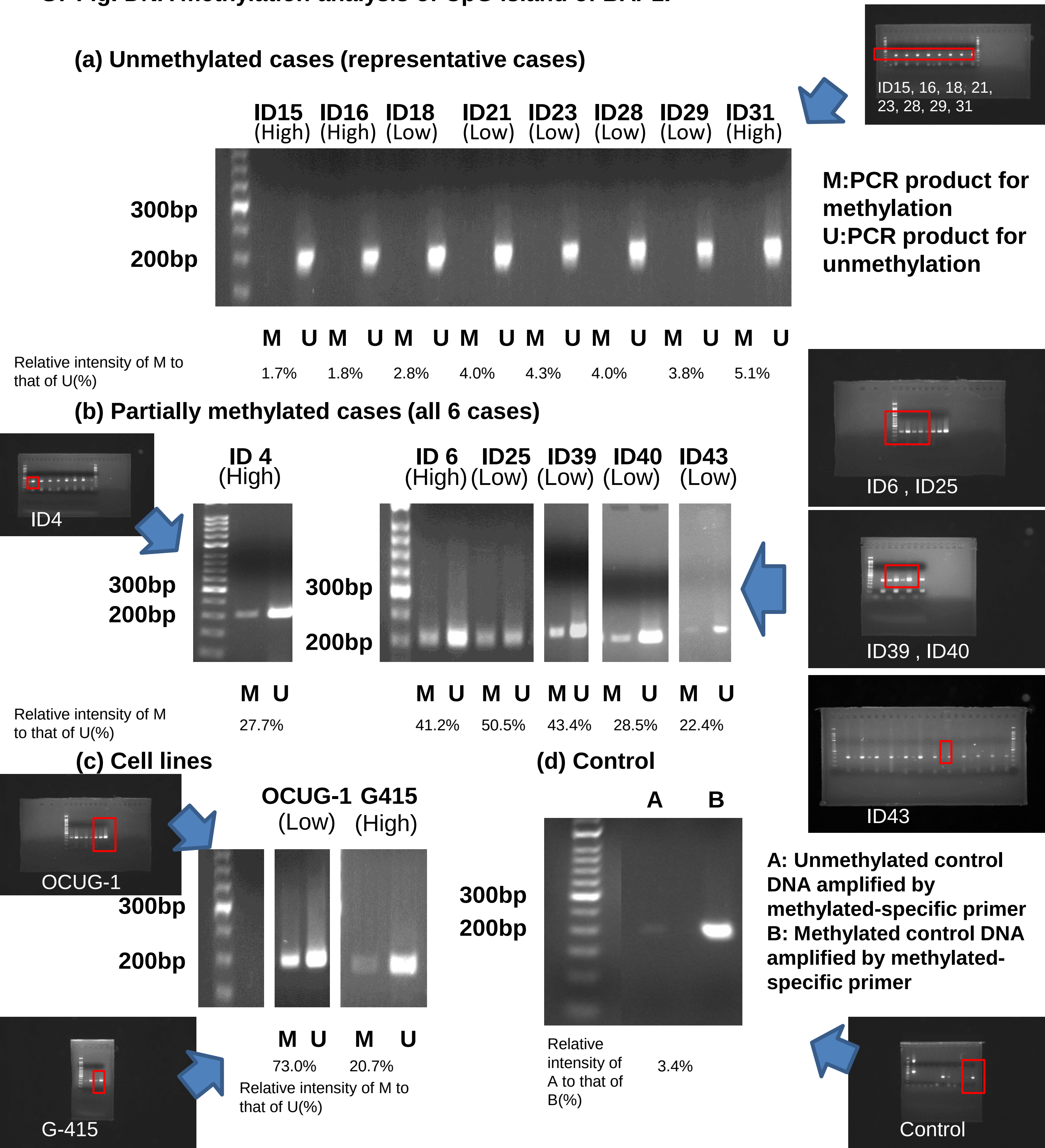

Supplement: S7 Fig — (PDF) [file pone.0206643.s011.pdf]
